# Polyfluorene-based white light conjugated polymers incorporating orange iridium(iii) complexes: the effect of steric configuration on their photophysical and electroluminescent properties†

**DOI:** 10.1039/c7ra11204a

**Published:** 2018-01-05

**Authors:** Jing Sun, Dongyu Wu, Long Gao, Minna Hou, Guojing Lu, Jie Li, Xinwen Zhang, Yanqin Miao, Hua Wang, Bingshe Xu

**Affiliations:** Key Laboratory of Interface Science and Engineering in Advanced Materials, Ministry of Education, Taiyuan University of Technology Taiyuan 030024 P. R. China sunjing@tyut.edu.cn wanghua001@tyut.edu.cn; Research Center of Advanced Materials Science and Technology, Taiyuan University of Technology Taiyuan 030024 P. R. China; Key Laboratory for Organic Electronics & Information Displays, Nanjing University of Posts and Telecommunications Nanjing 210023 P. R. China iamxwzhang@njupt.edu.cn

## Abstract

Different kinds of polyfluorene-based white light conjugated polymers with phosphorescent iridium(iii) complexes as orange emission groups and polyfluorene as blue emission groups were designed and synthesized. On the basis of adjusting substituent positions on iridium(iii) complexes, the conjugated polymers exhibited different steric configurations, *i.e.* hyperbranched and linear structures, and the PL emission peaks of iridium(iii) complexes had a significant change. Compared to linear conjugated polymers, hyperbranched white light conjugated polymers showed the best thermal stability and film forming properties. The white light single-emissive-layer devices with simplified configuration were also prepared in a wet process. All these devices realized good electroluminescence, especially the hyperbranched conjugated polymers in which the roll off phenomenon at high current density was effectively suppressed. Furthermore, EL spectra of hyperbranched polymers exhibited good stability at different driving voltages. A maximum luminance of 2469 cd m^−2^, a maximum current efficiency of 1.73 cd A^−1^ and the commission internationale de l'Eclairage (CIE) coordinates of (0.25, 0.23) showed white light was achieved from the HPF-Ir10 devices.

## Abstract 2

Owing to large steric hindrance, white light hyperbranched conjugated polymers exhibited the best electroluminescent performance with suppression of triplet–triplet annihilation.

## Graphical abstract

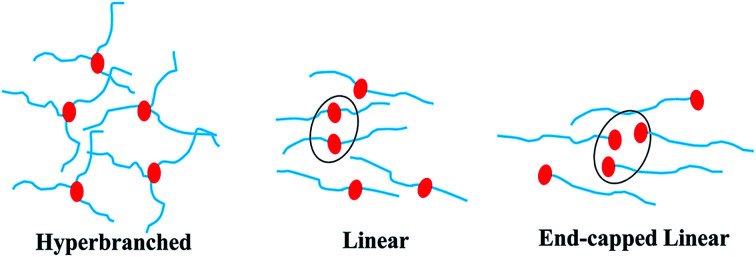

## 1. Introduction

White polymer light-emitting devices (WPLEDs)^1,2^ had attracted broad research interest due to their potential applications in large-area flexible displays and solid-state lighting sources, especially the phosphorescent polymers that could utilize both singlet and triplet excitons.^3–5^ The WPLEDs could be prepared from blended systems with different phosphorescent chromophores, however, serious phase separation existed that decreased the electroluminescent (EL) performance.^6–9^ In order to overcome this problem, single molecular systems of white-light polymers were designed and synthesized,^10,11^ and the possible approach was covalently incorporating modulated ratio of low band-gap chromophores into the blue-emitting polymers to achieve the white light emission.

As known that, polyfluorene (PF) were the very promising materials for blue-light emitting materials because of their high photoluminescence (PL) efficiency and good thermal stability which could be used as both the host and blue emitter^12,13^ in the single white light polymers system. Recently, the steric configuration of PF derivates^14–16^ in the single white light conjugated polymers were investigated to improve the electroluminescent (EL) properties. There were two main structure of the conjugated polymers, *i.e.* linear structure and hyperbranched structure. Y. Cao group had designed and synthesized a series of linear conjugated polymer with the phosphorescent chromophores incorporating to the PF main chain realizing the white light emission.^17,18^ However, the EL performance were unsatisfactory that the EL spectra at different driving voltages were not stable and the roll off phenomenon were serious at high current density. C. L. Yang group^19^ reported a novel phosphorescent white-light polymer with star-shaped structure utilized a triphenylamine-based iridium(iii) dendritic complexes as orange-emitting core and PF chains as blue-emitting arms, but more host arms were not good for charge transfer in the single polymers. Our group had synthesized a kind of hyperbranched white light polymer with tris[1-phenylisoquinolinato-C2,N]iridium(iii) (Ir(piq)_3_) as the red core and PF as the blue branches which took full advantages of the excitons. But the core of Ir(piq)_3_ were difficult to synthesize which had improved the preparation cost.^20^ Therefore, to obtain an appropriate steric configuration with high-quality iridium(iii) complexes were very important for the single white light polymers.

In this paper, we chose heteroleptic bis(1-phenylisoquinolinato)[3-(2-pyridyl)-5-phenyl-1,2,4-triazole] iridium(iii) [Ir(piq)_2_(pytzph)] as orange emission groups and PF as blue backbone to prepare the single white light conjugated polymers. By adjusting the substituent position on the ligand of Ir(piq)_2_(pytzph), three kind of white light conjugated polymers with different steric configuration (*i.e.* hyperbranched structure, linear structure and end-capped linear structure) were synthesized. In addition, the effect of steric configuration on their thermal stabilities, photophysical properties, film morphologies and EL performance were systematically and detailedly investigated. The results indicated that the WPLEDs from hyperbranched conjugated polymers with large steric hindrance had great potential in the organic photoelectric field.

## 2. Experimental section

### 2.1. Materials

All the raw materials were purchased and used without further purification. The solvents used for the synthesis of intermediates and end-products were purified by routine procedures under nitrogen protection. The orange iridium(iii) complexes utilized in this work were synthesized according to the literature.^21^

### 2.2. Synthetic procedures of polymers

#### 2.2.1. Hyperbranched polymer of HPF-Ir50

The reactive monomers of M1 (642.6 mg, 1 mmol), M2 (540.2 mg, 0.985 mmol) and M3 (10.59 mg, 0.01 mmol) were added into a 50 mL reaction flask with toluene (15 mL), and stirred for 30 min under nitrogen atmosphere at 25 °C. Then the catalyst of Pd(PPh_3_)_4_ (2 mol%), methyl trioctyl ammonium chloride (Aliquat 336) (15 mg) and aqueous solution of 2 mol L^−1^ K_2_CO_3_ (10 mL) were added into the mixture solution. The mixture were reacted at 90 °C for 60 h. Subsequently, the end-capping materials of benzeneboronic acid (20 mg) and bromobenzene (0.2 mL) were added in turn and further reacted for 12 h. After that, the mixture solution was washed by water, dried and poured into the methanol solution and the small molecules and catalyst residue in the precipitates were removed by soxhlet extraction. Finally, the crude products were purified by column chromatography, yield: 56%. ^1^H NMR: (600 MHz, CDCl_3_): *δ* (ppm) = 7.87 (1H, Ar–H), 7.76–7.67 (2H, Ar–H), 2.15 (2H, CH_2_), 1.23–1.09 (10H, 5 CH_2_), 0.89–0.78 (5H, CH_2_, CH_3_). ^13^C NMR: (600 MHz, CDCl_3_): *δ* (ppm) = 154.97, 143.52, 142.89, 131.81, 129.78, 128.88, 124.31, 122.84, 58.28, 43.09, 34.73, 32.82, 32.19, 26.68, 25.52, 16.79. Elemental analysis calcd (%) for C, 88.95; H, 10.18; N, 0.11. Found (%): C, 87.29; H, 9.98; N, 0.15.

#### 2.2.2. Hyperbranched polymer of HPF-Ir10

M1 (642.6 mg, 1 mmol), M2 (546.8 mg, 0.997 mmol) and M3 (2.12 mg, 0.002 mmol), yield: 52%. ^1^H NMR: (600 MHz, CDCl_3_): *δ* (ppm) = 7.84 (1H, Ar–H), 7.73–7.64 (2H, Ar–H), 2.12 (2H, CH_2_), 1.25–1.01 (10H, 5CH_2_), 0.89–0.74 (5H, CH_2_, CH_3_). ^13^C NMR: (600 MHz, CDCl_3_): *δ* (ppm) = 154.96, 143.54, 142.94, 131.73, 129.82, 128.99, 124.28, 122.86, 58.32, 43.14, 34.69, 32.76, 32.28, 26.75, 25.49, 16.98. Elemental analysis calcd (%) for C, 89.16; H, 10.24; N, 0.02. Found (%): C, 89.39; H, 10.57; N, 0.05.

#### 2.2.3. Hyperbranched polymer of HPF-Ir5

M1 (642.6 mg, 1 mmol), M2 (547.6 mg, 0.9985 mmol) and M4 (1.06 mg, 0.001 mmol), yield: 49%. ^1^H NMR: (600 MHz, CDCl_3_): *δ* (ppm) = 7.84 (1H, Ar–H), 7.73–7.63 (2H, Ar–H), 2.13 (2H, CH_2_), 1.26–1.04 (10H, 5CH_2_), 0.90–0.73 (5H, CH_2_, CH_3_). ^13^C NMR: (600 MHz, CDCl_3_): *δ* (ppm) = 154.91, 143.49, 142.90, 131.71, 129.83, 128.97, 124.26, 122.82, 58.30, 43.13, 34.65, 32.73, 32.26, 26.73, 25.46, 16.91. Elemental analysis calcd (%) for C, 89.20; H, 10.25; N, 0.01. Found (%): C, 87.98; H, 10.39; N, 0.11.

#### 2.2.4. Linear polymer of LPF-Ir10

M1 (642.6 mg, 1 mmol), M2 (547.3 mg, 0.998 mmol) and M3 (1.96 mg, 0.002 mmol), yield: 49%. ^1^H NMR: (600 MHz, CDCl_3_): *δ* (ppm) = 7.84 (1H, Ar–H), 7.73–7.64 (2H, Ar–H), 2.12 (2H, CH_2_), 1.25–1.01 (10H, 5CH_2_), 0.89–0.72 (5H, CH_2_, CH_3_). ^13^C NMR: (600 MHz, CDCl_3_): *δ* (ppm) = 154.52, 143.67, 142.54, 131.71, 129.83, 128.84, 124.43, 122.97, 58.07, 43.29, 34.70, 32.86, 32.12, 26.75, 25.08, 16.94. Elemental analysis calcd (%) for C, 89.16; H, 10.24; N, 0.02. Found (%): C, 89.50; H, 10.51; N, 0.08.

#### 2.2.5. Linear polymer of LPF-Ir5

M1 (642.6 mg, 1 mmol), M2 (547.6 mg, 0.999 mmol) and M4 (0.98 mg, 0.001 mmol), yield: 47%. ^1^H NMR: (600 MHz, CDCl_3_): *δ* (ppm) = 7.84 (1H, Ar–H), 7.73–7.64 (2H, Ar–H), 2.12 (2H, CH_2_), 1.25–1.02 (10H, 5CH_2_), 0.90–0.73 (5H, CH_2_, CH_3_). ^13^C NMR: (600 MHz, CDCl_3_): *δ* (ppm) = 154.52, 143.49, 142.71, 131.84, 129.85, 128.82, 124.39, 122.97, 58.51, 43.18, 34.45, 32.77, 32.12, 26.90, 25.42, 17.02. Elemental analysis calcd (%) for C, 89.20; H, 10.25; N, 0.01. Found (%): C, 87.94; H, 10.33; N, 0.08.

#### 2.2.6. End-capped linear polymer of ELPF-Ir5

M1 (642.6 mg, 1 mmol), M2 (548.2 mg, 0.9995 mmol) and M4 (0.9 mg, 0.001 mmol), yield: 53%. ^1^H NMR: (600 MHz, CDCl_3_): *δ* (ppm) = 7.83 (1H, Ar–H), 7.73–7.64 (2H, Ar–H), 2.12 (2H, CH_2_), 1.21–1.06 (10H, 5CH_2_), 0.85–0.76 (5H, CH_2_, CH_3_). ^13^C NMR: (600 MHz, CDCl_3_): *δ* (ppm) = 154.53, 143.44, 142.77, 131.92, 129.83, 128.84, 124.19, 122.97, 58.53, 43.23, 34.49, 32.84, 32.11, 26.96, 25.62, 17.11. Elemental analysis calcd (%) for C, 89.20; H, 10.25; N, 0.01. Found (%): C, 88.22; H, 10.18; N, 0.12.

### 2.3. Measurements

^1^H NMR spectra were measured using Bruker DXR 600 MHz spectrometers. The molecular weight were determined by waters GPC 2410 utilized THF solution as eluent. The concentration of samples was 0.2 mg mL^−1^ with a flow rate of 1 mL min^−1^ and the column calibration was polystyrene. Thermogravimetric analysis (TGA) curve were recorded on a Netzsch TG 209 F3 and differential scanning calorimetry (DSC) curve were measured on TA Q2000 that the heating rate were both 10 °C min^−1^ under nitrogen flow. Cyclic voltammetry (CV) data were measured on the Autolab/PG STAT302 electrochemical work station in acetonitrile (0.1 mol L^−1^) solution of tetrabutylammonium perchlorate as electrolyte at a scan rate of 50 mV s^−1^ at room temperature under nitrogen atmosphere. UV-vis absorption spectra were recorded on a Hitachi U-3900 spectrophotometer. PL spectra were recorded on a Fluoromax-4 spectrophotometer. The solution samples were added into quartz cuvette (12.5 mm × 12.5 mm × 45 mm) and the 1.5 nm slit widths were chosen; the film were spin-coated on the quartz plates (12 mm × 12 mm × 1 mm) and the 1.2 nm slit widths were chosen. The PL quantum yields (QYs) were measured using an IS080 Lab Sphere integrating sphere with 365 nm excitation. The fluorescence lifetime (*τ*) were measured by Edinburgh FLS 980 with 365 nm excitation by xenon lamp. The QYs and lifetime samples used in the tests were obtained as same as PL spectra in films. The film morphologies were observed by SPA-300HV atomic force microscopy (AFM).

### 2.4. Device fabrication and measurements

The device configuration were ITO/PEDOT:PSS (40 nm)/polymers (60 nm)/TPBi (40 nm)/LiF (1 nm)/Al (100 nm) and the devices were fabricated by wet process. First, ITO patterned glass substrates were pre-cleaned in turn by acetone, isopropanol and deionized water. Second, PEDOT:PSS layer was spin-coated on the ITO glass substrates and annealed at 120 °C for 20 min to remove the solvent. Third, polymers dissolved in chlorobenzene (12 mg mL^−1^) were separately spin-coated onto the uniform PEDOT:PSS layer and annealed at 100 °C for 15 min. Finally, TPBi layer, LiF layer and Al layer were deposited to the polymer layer in turn utilizing vacuum deposition. The current–voltage–luminescence characteristics of the devices were recorded using a combination of a Keithley source meter (model 2602) and a luminance meter. The EL spectra and commission internationale de l'Eclairage (CIE) coordinates of the devices were analyzed with a spectra-scan PR655 spectrophotometer. The external quantum efficiency (EQE) values were calculated according to ref. 22,23.

## 3. Results and discussion

### 3.1. Synthesis and characterization

**Scheme 1:**
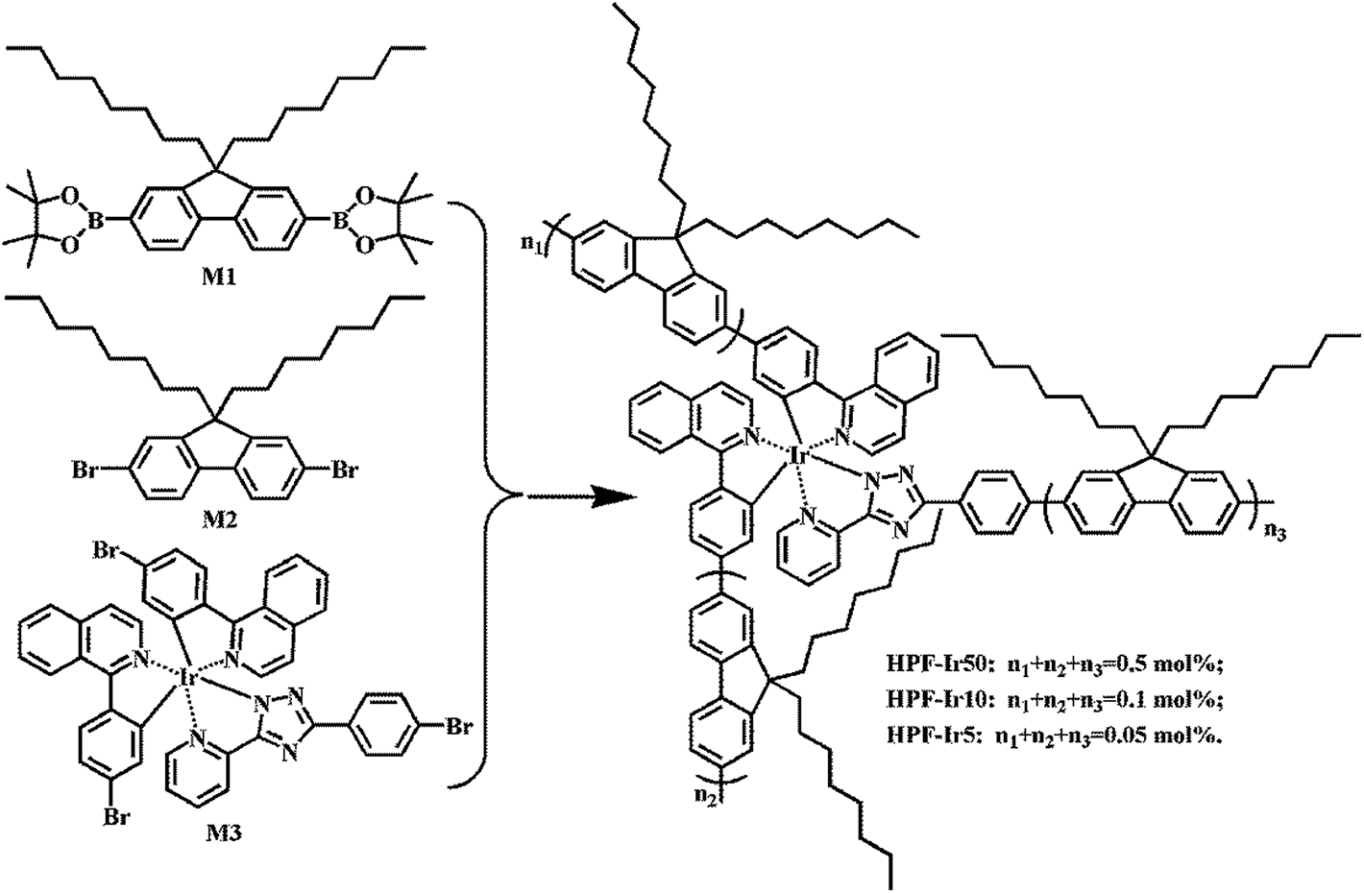
The synthetic route of white-light hyperbranched conjugated polymers.

**Scheme 2:**
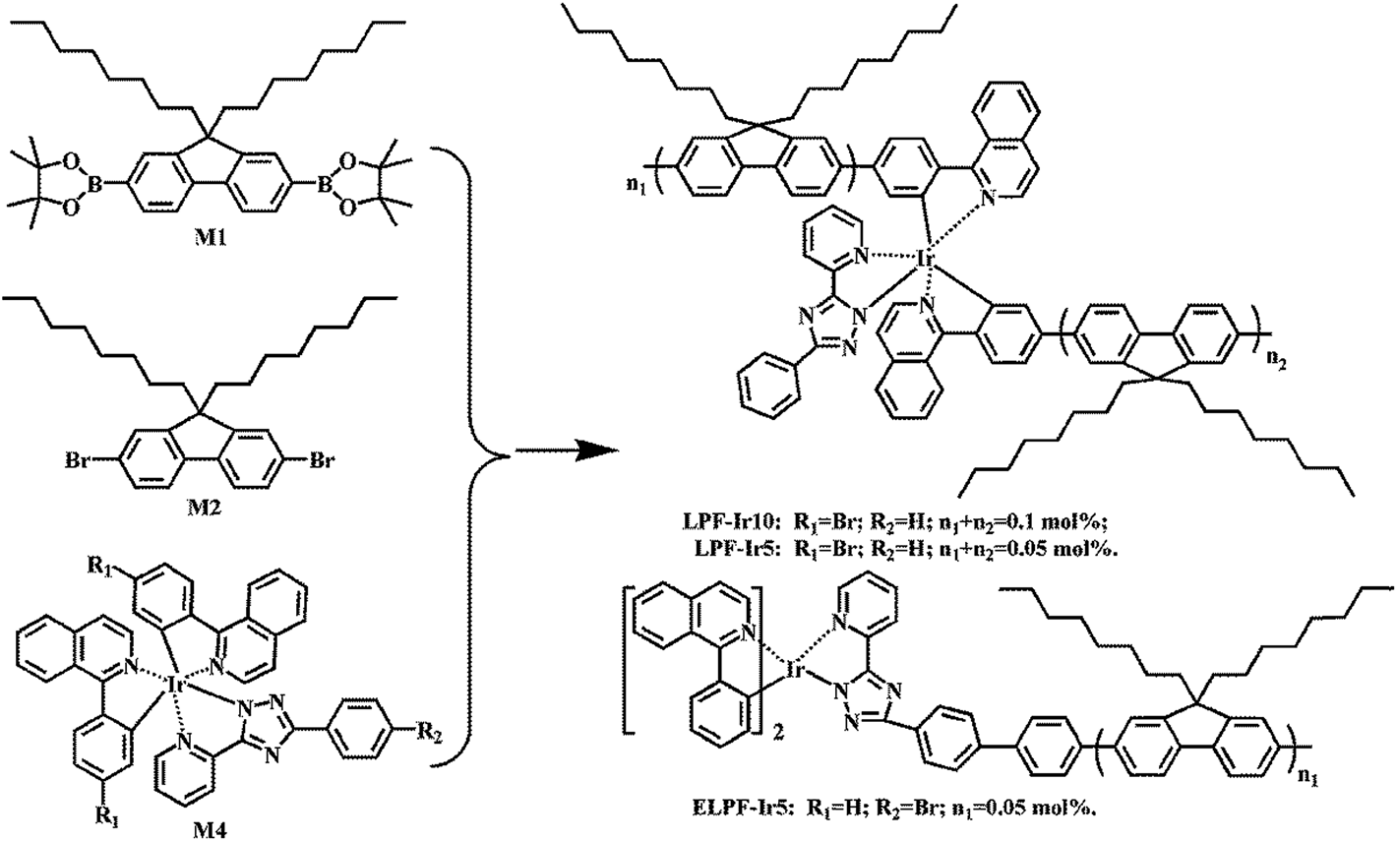
The synthetic route of white-light linear conjugated polymers.

The synthetic route of conjugated polymers were illustrated in Scheme 1 (hyperbranched structure) and Scheme 2 (linear structure). The orange iridium(iii) complexes Ir(piq)_2_(pytzph) were combined with PF segments through the covalent bond on the main or ancillary ligands, and the content of Ir(piq)_2_(pytzph) were from 0.05 to 0.5 mol%. The structure of different polymers were characterized by ^1^H NMR spectra. The number-average molecular weights (*M*_n_) and weight-average molecular weights (*M*_w_) of the polymers were measured by GPC and the polydispersity indices (PDIs) were obtained by *M*_w_/*M*_n_. The *M*_n_ and *M*_w_ were from 9500 g mol^−1^ to 17 400 g mol^−1^ and from 27 300 g mol^−1^ to 42 800 g mol^−1^, respectively, with the PDIs of 2.25–2.88 proving the narrow distribution of molecular weight that guarantee the stability of the polymers in the application. The thermal properties were studied by TGA curves and DSC curves under the protection of nitrogen and the detailed data were shown in Table 1. The polymers had good thermal stability that the thermal decomposition temperature (*T*_d_) exceeded 390 °C and the glass-transition temperature (*T*_g_) over 110 °C. By contrast, the hyperbranched polymers exhibited better thermal stability owing to the large steric structure which were in favor of forming excellent polymer films and realizing stability in the fabrication process of WPLEDs.

**Table 1:** The molecular weights and thermal stability of conjugated polymers

Polymers	*M*_n_ (g mol^−1^)	*M*_w_ (g mol^−1^)	PDIs	Feed ratio (mol%)	Real content (mol%)	*T*_d_ (°C)	*T*_g_ (°C)
HPF-Ir50	15.6 × 10^3^	35.0 × 10^3^	2.25	0.5	0.237	400	145
HPF-Ir10	9.5 × 10^3^	27.3 × 10^3^	2.88	0.1	0.048	418	—
HPF-Ir5	13.6 × 10^3^	36.7 × 10^3^	2.69	0.05	0.038	399	153
LPF-Ir10	11.7 × 10^3^	31.1 × 10^3^	2.67	0.1	0.046	417	131
LPF-Ir5	12.3 × 10^3^	31.7 × 10^3^	2.58	0.05	0.033	394	115
ELPF-Ir5	17.4 × 10^3^	42.8 × 10^3^	2.47	0.05	0.041	404	149

### 3.2. Photophysical properties

**Fig. 1:**
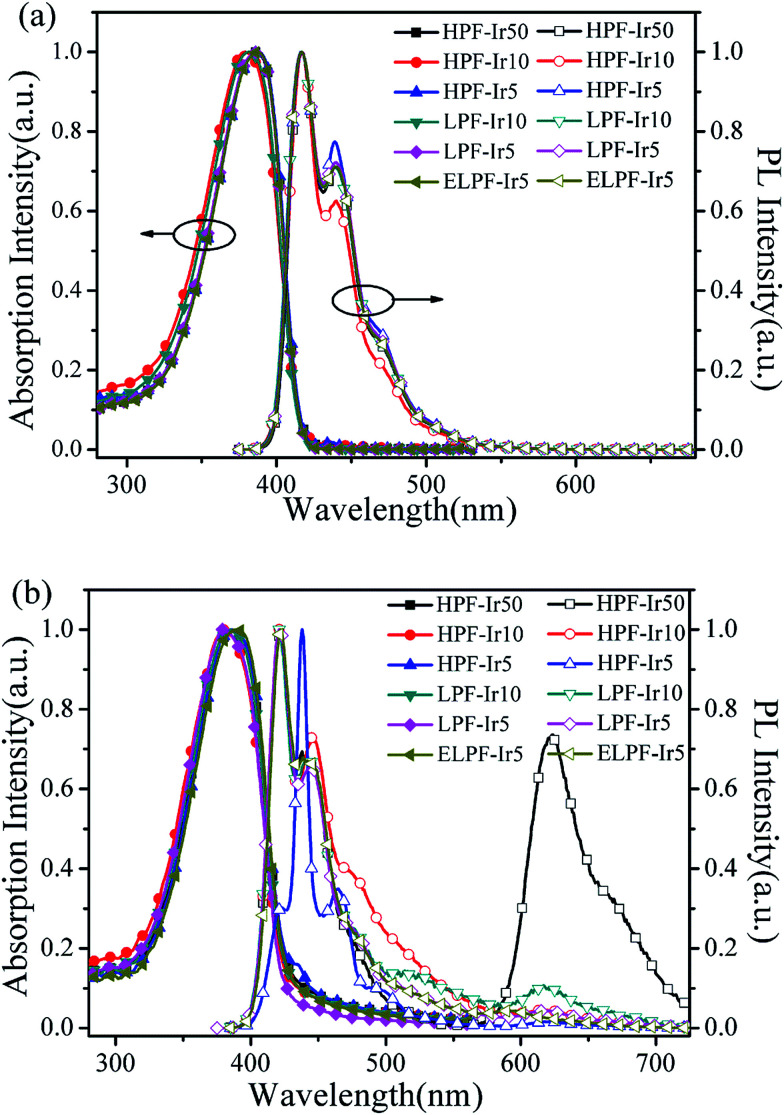
UV-vis spectra and PL spectra of the conjugated polymers in CHCl_3_ solution (a) and films (b).

**Fig. 2:**
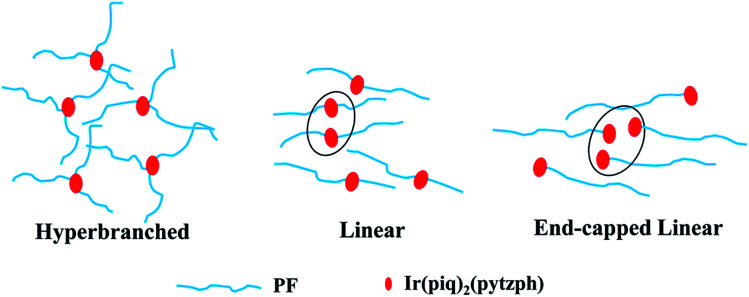
The molecular accumulation schematic of the conjugated polymers.

Fig. 1 processed UV-vis spectra and PL spectra of the conjugated polymers in solution and films. In solution, the absorption band were located at 387 nm attributing to the π–π* transition of PF backbone and the emission band was at 416, 439 and 469 nm belong to the emission of PF backbone. No obvious orange emission were found. By contrast, the absorption band in films were similar to the absorption band in solution and the detailed data were shown in Table 2. The first blue emission peaks had red shift owing to the aggregation^24^ that enhanced the intersystem crossing between the adjacent molecules and the emission peak at 440 nm had not changed because of the formation of β phase.^25,26^ Furthermore, the orange emission from the conjugated polymers in films appeared, however, the emission peaks were different in the three steric configuration, especially the ELPF-Ir5 that its orange emission peak had a large blue shift compared to the others. It could be attributed to the auxiliary ligand of 1,2,4-triazole derivatives^27^ combined with the conjugated PF that decreased the HOMO energy level and increased the energy gap between the HOMO and LUMO energy level of the incorporated iridium(iii) complexes in the polymers.^28,29^ In the hyperbranched polymers, though the main and auxiliary ligands were both combined with PF, the main ligands were dominant to the emission of iridium(iii) complexes. The fluorescence quantum yields (QYs) and the lifetime (*τ*) of orange emission from conjugated polymer films were recorded in Table 2. It was noted that, in hyperbranched structure, the QYs were improved with increasing the content of iridium(iii) complexes, but reduced with the increasing content largely because of the higher concentration which would induced the triplet–triplet annihilation.^12^ The molecular aggregation schematic of the conjugated polymers were shown in Fig. 2. The hyperbranched structure had large steric hindrance and the orange cores were fully isolated by PF branches, however, both the linear structure could not effectively suppress the collision of iridium(iii) complexes that would result in the serious triplet–triplet annihilation.

**Table 2:** The thermal stability, photophysical and electrochemical data of the conjugated polymers

Polymers	In solution^a^ (nm)		In film^b^ (nm)		QY^b^ (%)	*τ* c (ns)	HOMO (eV)	LUMO (eV)	*E*_g_ (eV)
	*λ*_abs_	*λ*_PL_	*λ*_abs_	*λ*_PL_					
HPF-Ir50	385	416/441/469	385	421/439/469/622	13.60	1656	−5.79	−2.91	2.88
HPF-Ir10	379	417/440/469	379	421/445/474/617	20.82	1536	−5.75	−2.86	2.89
HPF-Ir5	387	416/439/470	386	422/438/464/617	16.49	1554	−5.77	−2.90	2.87
LPF-Ir10	381	416/439/469	385	321/442/472/617	21.02	1480	−5.76	−2.88	2.88
LPF-Ir5	386	416/440/469	379	421/444/476/616	19.45	1358	−5.78	−2.88	2.90
ELPF-Ir5	387	416/439/469	389	422/441/470/592	24.60	938	−5.75	2.88	2.87

a In diluted CHCl_3_ solution of 10^−6^ mol L^−1^.

b Coated on quartz plate by spin-coating process from 12 mg mL^−1^ toluene solution under 365 nm excitation.

c The average lifetimes measured the orange emission peaks excited at 365 nm in films.

**Fig. 3:**
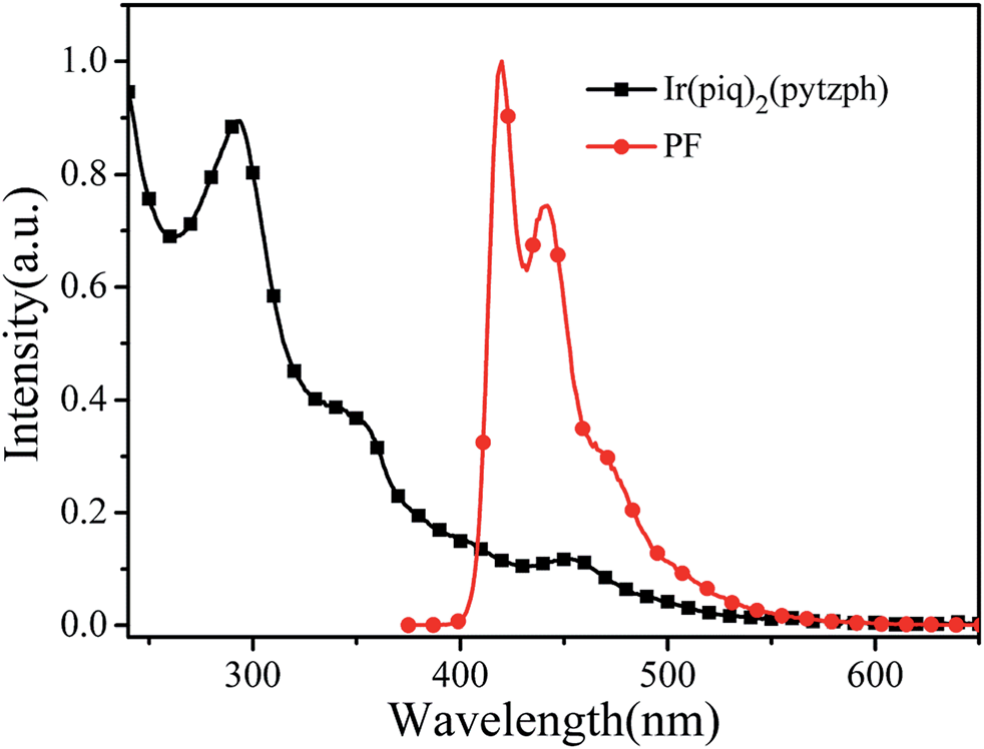
UV-vis spectrum of Ir(piq)_2_(pytzph) and PL spectrum of PF in the diluted CHCl_3_ solution.

**Fig. 4:**
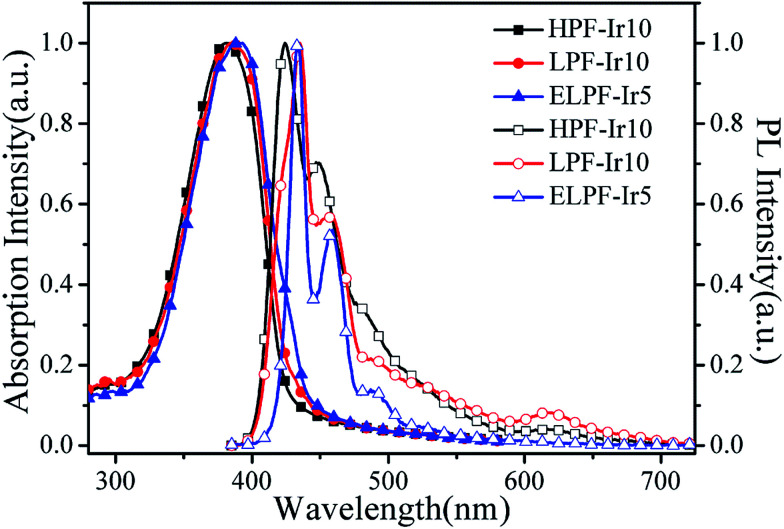
The UV-vis absorption spectra and PL spectra of HPF-Ir10, LPF-Ir10 and ELPF-Ir5 in films annealed at 100 °C.

In order to investigate the energy transfer process from the blue PF segments to the orange Ir(piq)_2_(pytzph), PL spectrum of PF in 10^−6^ mol L^−1^ solution and UV-vis spectrum of Ir(piq)_2_(pytzph) in 10^−5^ mol L^−1^ solution were measured in Fig. 3. The absorption band of Ir(piq)_2_(pytzph) was overlapped with the emission band of PF between 410 nm and 540 nm that indicated the effective energy transfer from PF to Ir(piq)_2_(pytzph) and the white light emission could be obtained from these conjugated polymers.^30^ The detailed lifetime at orange emission from the conjugated polymer films were summarized in Table 2. It was noted that the lifetime of hyperbranched polymers were longer than the linear polymers because more branches incorporating the iridium(iii) complexes had enhanced the energy transfer from PF backbone to Ir(piq)_2_(pytzph). In addition, the lifetime at orange position of hyperbranched polymers were prolonged with the increased content of iridium(iii) complexes indicating that more effective energy transfer from PF to Ir(piq)_2_(pytzph) were realized at high concentration. The UV-vis spectra and PL spectra of films after annealing at 100 °C were also investigated (Fig. 4) to characterize the film stability. The absorption band and emission peaks of hyperbranched polymers had no obvious change, however, the linear polymers exhibited obvious change owing to the little steric hindrance.^31^

### 3.3. Thin film morphologies

**Fig. 5:**
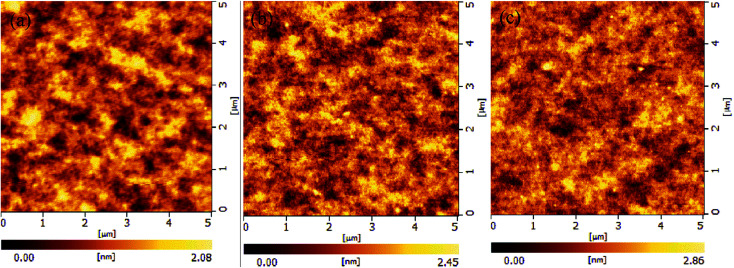
The AFM morphologies of the conjugated polymers HPF-Ir10, LPF-Ir10 and ELPF-Ir5 films coated on the ITO glasses (5 × 5 μm^2^).

The surface morphologies of the conjugated polymer films spin-coated on the PEDOT:PSS layer from 12 mg mL^−1^ chlorobenzene solution were characterized by AFM, as shown in Fig. 5. It was noted that the thin film surface of all polymers were very smooth and the root mean square (RMS) of HPF-Ir10, LPF-Ir10 and ELPF-Ir10 films were separately 0.34 nm, 0.38 nm and 0.41 nm. These results indicated that hyperbranched polymer had the best film-forming properties among the three kind of conjugated polymers which was in favor of preparing the single white-light emitting layer in the WPLEDs.^32,33^

### 3.4. Electroluminescent properties

**Fig. 6:**
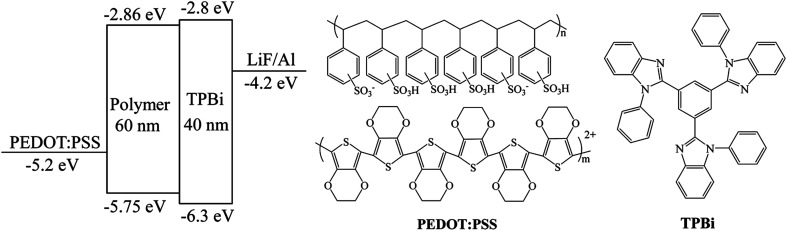
Schematic diagrams of the device configuration and the molecular structure of PEDOT:PSS and TPBi.

The device configuration of WPLEDs (ITO/PEDOT:PSS (40 nm)/polymers (60 nm)/TPBi (40 nm)/LiF (1 nm)/Al (100 nm)) and the used molecular structure were depicted in Fig. 6. It was clearly seen that the HOMO energy level of the conjugated polymers were about −5.75 eV that could match with the HOMO energy level of PEDOT:PSS; the LUMO energy level was about −2.86 eV that could match better with the LUMO energy level of electron-transporting TPBi layer (−2.8 eV). At the same time, the HOMO energy level of TPBi (−6.3 eV) could restrain the hole transferring to the TPBi layer that ensured the excitons recombination on the single white-light emitting layer.^34^

**Fig. 7:**
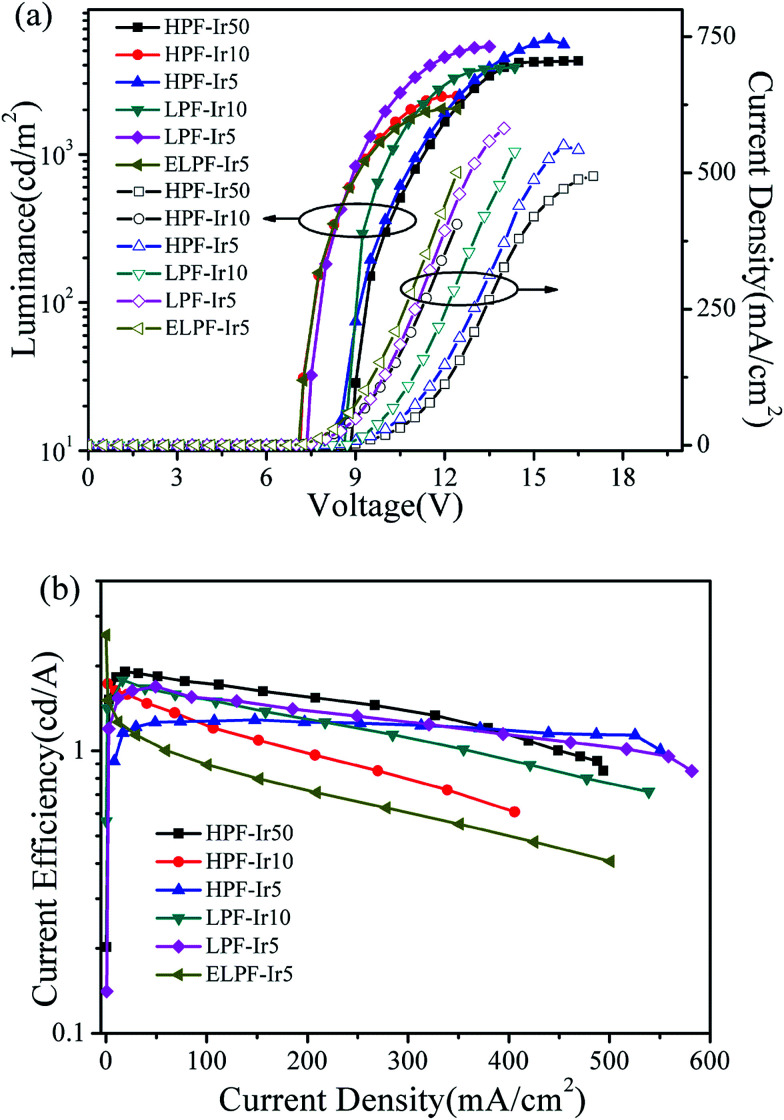
Luminance–voltage–current density (*L*–*V*–*J*) curves (a) and current density–current efficiency (*J*–CE) curves (b) of the conjugated polymers.

Fig. 7 expressed the luminance–voltage–current density (*L*–*V*–*J*) curves (a) and current density–current efficiency (*J*–CE) curves (b) of the conjugated polymers. It was noted that the conjugated polymers with different steric configuration had realized electroluminescence with the maximum luminance exceeded 2000 cd m^−2^ and the EQE (Table 3) were improved by increasing the content of iridium(iii) complexes. The WPLEDs from hyperbranched polymers exhibited the slighter “roll off” phenomenon owing to the triplet–triplet annihilation suppressed by the large steric hindrance (Fig. 7(b)). However, ELPF-Ir5 had a serious roll-off at high current density owing to the exposed iridium(iii) complexes at the end position in PF chain that enhanced the interaction of adjacent iridium(iii) complexes resulting in the triplet–triplet annihilation.

**Table 3:** The detailed EL data of the conjugated polymers

Polymers	*λ*_EL_ (nm)	*V*_turn-on_ a (V)	*L*_max_ (cd m^−2^)	CE_max_ (cd A^−1^)	EOE (%)	CIE^b^
HPF-Ir50	432/460/491/523/629	8.5	4276	1.91	1.87	(0.45, 0.28)
HPF-Ir10	430/454/484/518/626	7.2	2469	1.73	1.30	(0.25, 0.23)
HPF-Ir5	432/464/492/524/624	7.5	5976	1.29	0.83	(0.21, 0.24)
LPF-Ir10	433/459/491/524/619	8.2	3863	1.78	1.26	(0.23, 0.20)
LPF-Ir5	432/462/490/520/617	7.5	5343	1.69	1.18	(0.19, 0.20)
ELPF-Ir5	434/460/493/523/594	6.7	2036	2.58	1.48	(0.25, 0.27)

a Obtained at 1 cd m^−2^.

b At 9 V.

**Fig. 8:**
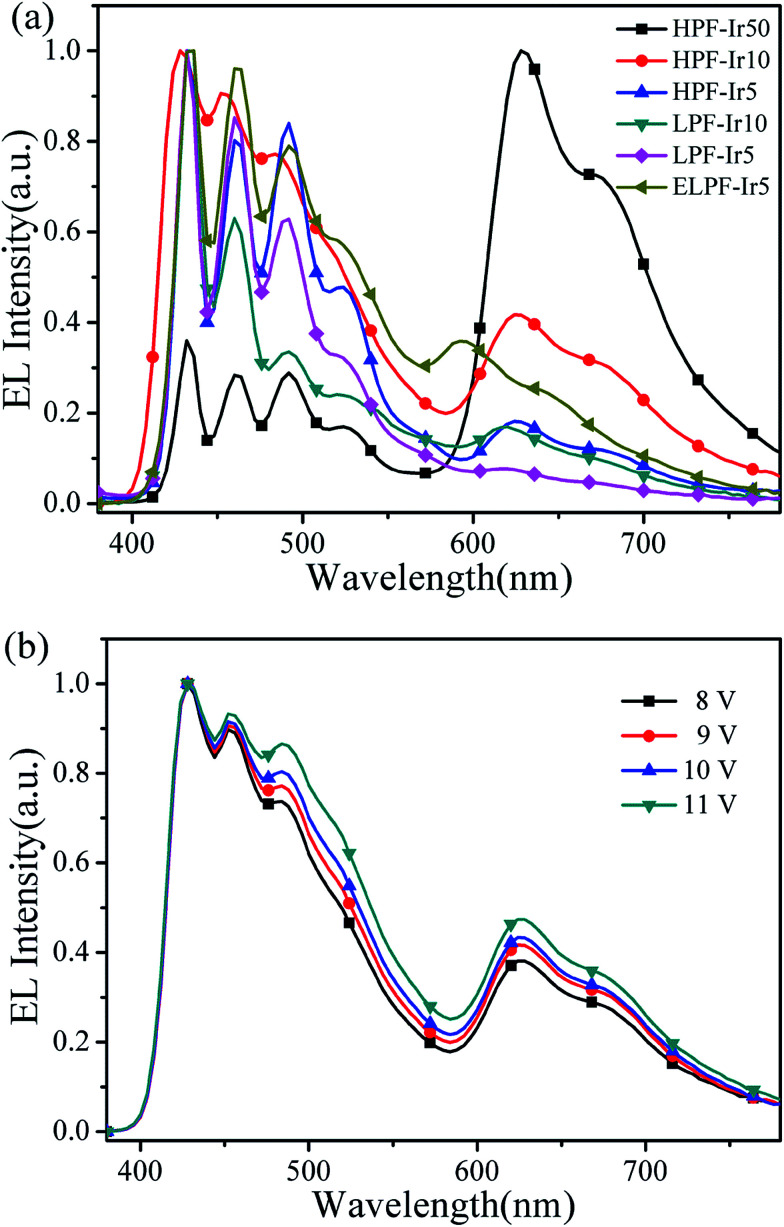
EL spectra of all conjugated polymers (a) and EL spectra of HPF-Ir10 at different driving voltages (b).

Furthermore, EL spectra of conjugated polymers were investigated as described in Fig. 8(a) and the CIE coordinates were summarized in Table 3. The blue emission peaks were located at about 434 nm, 460 nm and 491 nm belong to the emission of PF, and the orange emission peaks were separately at 626 nm (hyperbranched structure), 619 nm (linear structure) and 594 nm (end-capped linear structure). Compared to PL process, the orange emission in EL process had dramatically enhanced with a little red shift. It could be attributed to different mechanisms that the charge trapping of iridium(iii) complexes were dominated in EL process and the excitons recombination were enhanced, while the energy transfer from PF backbone to iridium(iii) complexes were dominated in PL process.^35^ In addition, the spectra stability of HPF-Ir10 at different driving voltages were recorded in Fig. 8(b). With the increasing driving voltages, EL spectra of HPF-Ir10 had little changes demonstrating the good EL stability of the hyperbranched polymers.

The abovementioned research results indicated that all conjugated polymers could be used to prepare the WPLEDs, however, the end-capped linear conjugated polymers with iridium(iii) complexes at the end position was easy to induce triplet–triplet annihilation at high current density. In contrast, the hyperbranched polymers exhibited the best EL performance. Further investigations on device optimization were ongoing to improve the device performance.

## 4. Conclusion

In summary, three kind of polyfluorene-based white light conjugated polymers with phosphorescent iridium(iii) complexes as orange emission were designed and synthesized which processed different steric configuration (hyperbranched and linear structure) with good thermal stability, photophysical properties and film forming properties. Compared to the linear polymers, the energy transfer from PF backbone to iridium(iii) complexes in hyperbranched conjugated polymers were most effective. The WPLEDs utilized the conjugated hyperbranched polymers as the single white light emitting layer by wet process exhibited better EL performance with slight roll off phenomenon with a maximum luminance of 2469 cd m^−2^, a maximum current efficiency of 1.73 cd A^−1^ and a CIE coordinate of (0.25, 0.23).

## Conflicts of interest

There are no conflicts to declare.

## Supplementary Material

RA-008-C7RA11204A-s001
