# Supplementary material for: Polyfluorene-based white light conjugated polymers incorporating orange iridium(iii) complexes: the effect of steric configuration on their photophysical and electroluminescent properties
Source: RSC Adv. 2018 Jan 5;8(3):1638–46. doi: 10.1039/c7ra11204a (PMC9077086; doi:10.1039/c7ra11204a)
Supplement: RA-008-C7RA11204A-s001 [file RA-008-C7RA11204A-s001.pdf]

Electronic Supplementary Material (ESI) for RSC Advances.
This journal is © The Royal Society of Chemistry 2017

Polyfluorene-based white light conjugated polymers incorporating orange iridium(Ⅲ) complexes: Effect of steric configuration on their photophysical and electroluminescent properties

Jing Sun[a,b,*], Dongyu Wu[a,b], Long Gao[a,b], Guojing Lu[a,b], Jie Li[a,b], Xinwen Zhang[c,**], Yanqin Miao[a,b], Hua Wang[a,b,***], Bingshe Xu[a,b]

[a] *Key Laboratory of Interface Science and Engineering in Advanced Materials, Taiyuan University of Technology, Ministry of Education, Taiyuan 030024, P. R. China. E-mail address: sunjing@tyut.edu.cn; wanghua001@tyut.edu.cn*

[b] *Research Center of Advanced Materials Science and Technology, Taiyuan University of Technology, Taiyuan 030024, P. R. China*

[c] *Key Laboratory for Organic Electronics & Information Displays, Nanjing University of Posts and Telecommunications, Nanjing 210023, P. R. China. E-mail address: iamxwzhang@njupt.edu.cn*

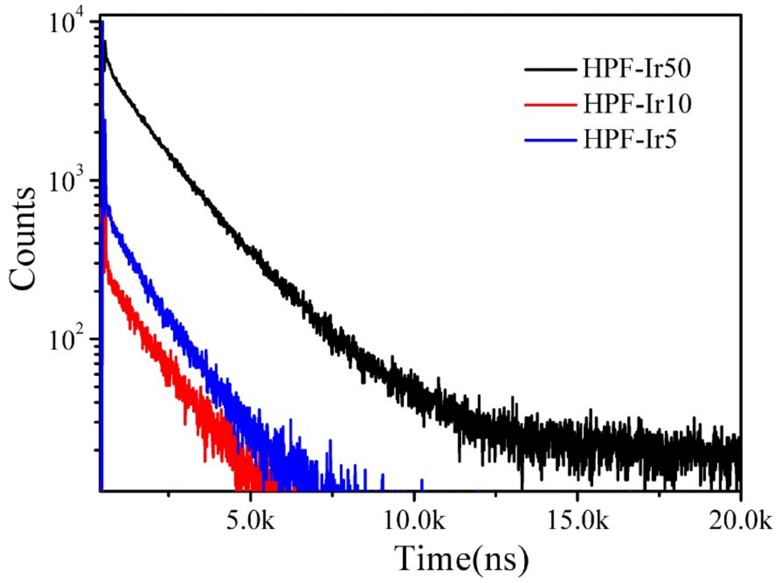

Fig. S1 The lifetime decay curves of the hyperbranched polymer films.

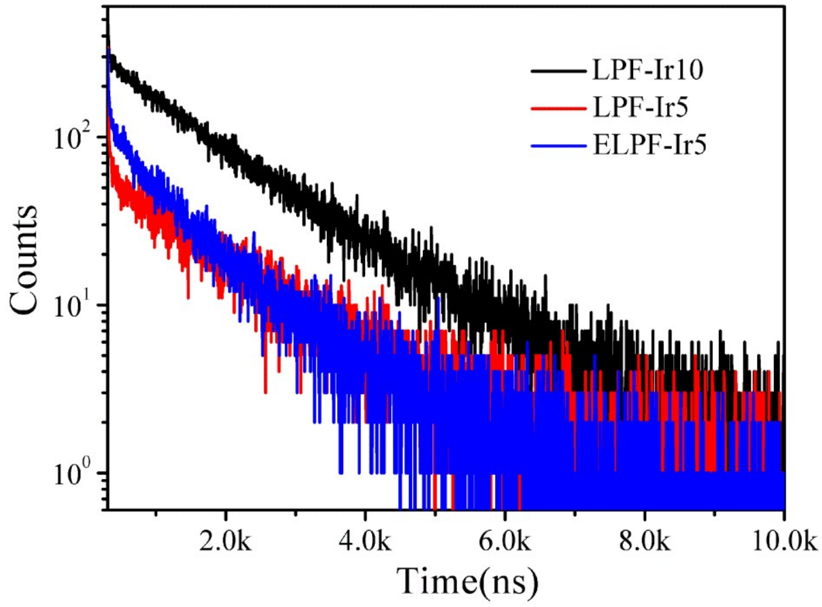

Fig. S2 The lifetime decay curves of the linear polymer films.

Table S1 The detailed data of fluorescence lifetime.

Polymers	τ_1(ns)	τ_2(ns)	χ^2	$\tau_{average}$(ns)
HPF-Ir50	562	1819	1.277	1656
HPF-Ir10	499	1674	1.026	1536
HPF-Ir5	561	1578	1.179	1554
LPF-Ir10	1480	-	1.098	1480
LPF-Ir5	1358	-	1.249	1358
ELPF-Ir5	938	-	1.153	938
